# Assessing peptic ulcer risk with the HAMPROW score in the general Chinese population

**DOI:** 10.1038/s41598-024-55224-0

**Published:** 2024-02-23

**Authors:** Binli Wang, Weitao Yu, Zheyu Zhang, Weili Jin, Haojun Chen, Linfeng Wang, Min Xu, Chaoqun Hou, Zhiquan Qian, Ziyue Qiu, Sheng Zhang

**Affiliations:** 1 Department of Neurology, Huzhou Nanxun People’s Hospital, Zhejiang Provincial People’s Hospital Nanxun District, Huzhou, China; 2 Center for Rehabilitation Medicine, Department of Neurology, Zhejiang Provincial People’s Hospital (Affiliated People’s Hospital), Hangzhou Medical College, Hangzhou, 310014 Zhejiang China; 3 The Second School of Clinical Medicine, Hangzhou Normal University, Hangzhou, Zhejiang China; 4 Department of Neurology, School of Medicine, The Second Affiliated Hospital of Zhejiang University, Hangzhou, China; 5 Department of Gastroenterology, Huzhou Nanxun People’s Hospital, Zhejiang Provincial People’s Hospital Nanxun District, Huzhou, China; 6 Department of Nephrology, Huzhou Nanxun People’s Hospital, Zhejiang Provincial People’s Hospital Nanxun District, Huzhou, China; 7 Department of Science and Education, Huzhou Nanxun People’s Hospital, Zhejiang Provincial People’s Hospital Nanxun District, Huzhou, China

**Keywords:** Gastroenterology, Medical research, Risk factors

## Abstract

The timely identification of individuals at high risk for peptic ulcers (PUs) is vital in preventing gastrointestinal bleeding after antiplatelet therapy. This study was designed to determine PU risk factors and develop a risk assessment model for PU detection in the general Chinese population. In a prospective dataset, clinical data from individuals undergoing gastroscopic evaluation between April 2019 and May 2022 were recorded. PUs were defined as mucosal defects exceeding 5 mm confirmed via gastroscopy. Participants were categorized into development (April 2019 to April 2021) and validation (May 2021 to May 2022) sets based on chronological order. LASSO-derived logistic regression analysis was employed to create a score, which was further validated via temporal validation. A total of 902 patients were ultimately enrolled, 204 (22.6%) of whom had PUs based on endoscopic findings. In the development cohort (n = 631), seven independent risk factors emerged: male sex (OR = 2.35, *P* = 0.002), white blood cell (WBC) count (OR = 1.16, *P* = 0.010), red blood cell (RBC) count (OR = 0.49, *P* < 0.001), globulin level (OR = 0.92, *P* = 0.004), albumin level (OR = 0.94, *P* = 0.020), pepsinogen I (PGI) level (OR = 1.01, *P* < 0.001), and positive *Helicobacter pylori* (HP) antibody (OR = 2.50, *P* < 0.001). Using these factors, a nomogram (HAMPROW score [hazard ratio (HP) antibody, albumin, male, PGI, RBC, globulin, and WBC]) was developed for individual PU prediction. The ability of the HAMPROW score to predict survival was confirmed with AUCs of 0.854 (95% CI 0.816–0.891) and 0.833 (95% CI 0.771–0.895) in the development and validation sets, respectively. In conclusion, the HAMPROW score can be used to screen for PUs effectively in the general Chinese population, facilitating personalized early detection of high risk of gastrointestinal bleeding before antiplatelet therapy.

## Introduction

Antiplatelet medications are currently the core treatment for atherosclerotic cardiovascular disease (ASCVD) prevention^1^, but they carry an increased risk of gastrointestinal bleeding. Studies have shown a 1.8-fold increased risk of gastrointestinal bleeding during low-dose aspirin (LDA) treatment and a 7.4-fold increased risk during dual antiplatelet therapy^2^. Moreover, epidemiological data have demonstrated that approximately 15–20% of patients receiving antiplatelet therapy^3,4^ have preexisting peptic ulcers (PUs), and a history of PUs can increase the risk of gastrointestinal bleeding by more than 6-fold^5^. However, it is rare for people to undergo PU risk screening before taking antiplatelet drugs. In most cases, people need to undergo physical examinations to determine the necessity of taking antiplatelet drugs. Identifying a simple tool that could screen for PU risk during routine clinical evaluation would be valuable for preventing gastrointestinal bleeding after antiplatelet therapy.

Currently, endoscopy is the most reliable method for screening PUs. However, its limited tolerance, high cost, and incompatibility with dynamic observation render it unsuitable for routine clinical evaluation, and it cannot be widely promoted as a screening tool for PUs in clinical practice. Studies have shown that the main mechanism of gastrointestinal injury induced by antiplatelet medications is local mucosal damage and the inhibition of mucosal repair^6^. In recent years, many studies have identified biomarkers that can predict PUs, such as platelet count, white blood cell (WBC) count, pepsinogen I (PGI), and gastrin 17 (G-17)^7–9^. However, most related studies have focused on specific clinical and hematological markers, resulting in limited value in identifying PUs using a single marker. Moreover, comparisons between the markers are lacking, and some of the markers reflect the same underlying mechanism of PU occurrence. Therefore, it may be more rational to endeavor to extract and integrate these markers for PU screening.

Nomograms, a pictorial representation of a complex mathematical formula (Grimes, 2008), reflect various factors simultaneously to help patients visualize their probability of developing a disease^10^. Several prediction nomogram models have been developed to effectively predict bleeding in PUs^11,12^, but studies focusing on screening for PUs in the physically examined population are rare. Therefore, this study was designed to develop and validate a nomogram for individually predicting PUs in the physically examined population, providing a reliable clinical tool for the early identification of PUs. This study provides insights into safe personalized treatment for potential recipients of antiplatelet therapy.

## Methods

### Study design and study population

From April 2019 to May 2022, data were prospectively and continuously collected from participants who underwent upper gastrointestinal mucosal assessment as part of their physical examination at our hospital. The inclusion criteria for patients were as follows: (i) ≥ 18 years of age, (ii) no severe organ failure, and (iii) no indication of hospitalization for gastrointestinal symptoms. The exclusion criteria for patients were as follows: (i) had specific gastrointestinal diseases, including acute upper gastrointestinal bleeding and esophageal malignant tumors; (ii) exhibited intolerance during endoscopy; (iii) poor image quality preventing proper assessment; (iv) had a history of upper gastrointestinal surgery and an upper gastrointestinal mucosal assessment process that was prospectively formulated; and (v) no documented history of proton-pump inhibitor (PPI) usage or cessation of such medications for a duration exceeding one month. The process involved collecting blood samples for PG, gastrin 17 (G-17) and *Helicobacter pylori* (HP) antibody analysis and gastroscopic assessment of gastrointestinal mucosal morphology.

### Patient involvement

Participants and their families were not involved in setting the research question or the outcome measures, but they were intimately involved in the design and implementation of the research. Participants and their families were also central to dissemination of the baseline information, thereby helping the study to proceed.

### Ethics statement

This retrospective study was approved by the human ethics committee of Huzhou Nanxun District People's Hospital (No. 2017N016). All subjects provided written informed consent prior to the study, and the protocols were approved by the local ethics committee. All clinical investigations were conducted according to the principles expressed in the Declaration of Helsinki.

### Clinical data extraction

The following data were extracted for each patient: age, sex, past medical history (hypertension, diabetes, and stroke), medication history (antiplatelet drugs, anticoagulants, and nonsteroidal anti-inflammatory drugs (except for aspirin), atherosclerotic cardiovascular disease (ASCVD) risk, and laboratory test results. All laboratory test results were acquired within 24 h before endoscopic examination (routine blood test results, liver function, coagulation function, electrolytes, blood lipids, PGI, PGII, G-17, HP antibody). The China-PAR^13^ model, which is specifically tailored for the Chinese population, was utilized to assess the risk of ASCVD. High-risk ASCVD was defined as having a 10-year ASCVD risk ≥ 10% or a lifetime risk (the cumulative probability of developing cardiovascular and cerebrovascular diseases from the current age until reaching 85 years) ≥ 32.8%. The ASCVD risk was evaluated through the following link: https://www.cvdrisk.com.cn/ASCVD/Eval.

### Endoscopy procedure for mucosal assessment

The upper gastrointestinal mucosal assessment process was prospectively formulated. The physically examined patients underwent endoscopy examination after one night of fasting. Four endoscopists participated in this study and were responsible for reviewing the images to determine the presence of PUs (at least 3 years of experience in upper gastrointestinal endoscopy with more than 3000 examinations). All the examinations were carried out according to strict protocols. A total of 40 films of the whole stomach and 8 films of the whole duodenum were taken via endoscopy. Blood samples were collected for PG, gastrin 17 (G-17) and *Helicobacter pylori* (HP) antibody analysis, and gastroscopic assessment of gastrointestinal mucosal morphology was performed.

### The assessment of PU

The upper gastrointestinal mucosal damage was assessed by the modified Lanza score (MLS)^14^. A score of 5 indicated that the diameter of the mucosal defect was > 5 mm, which was defined as a gastric ulcer (GU). Duodenal ulcers (DUs) were defined as mucosal defects > 5 mm in diameter. PUs were defined as GUs (MLS = 5 points) and/or DUs.

### Sample size

**Figure 1:**
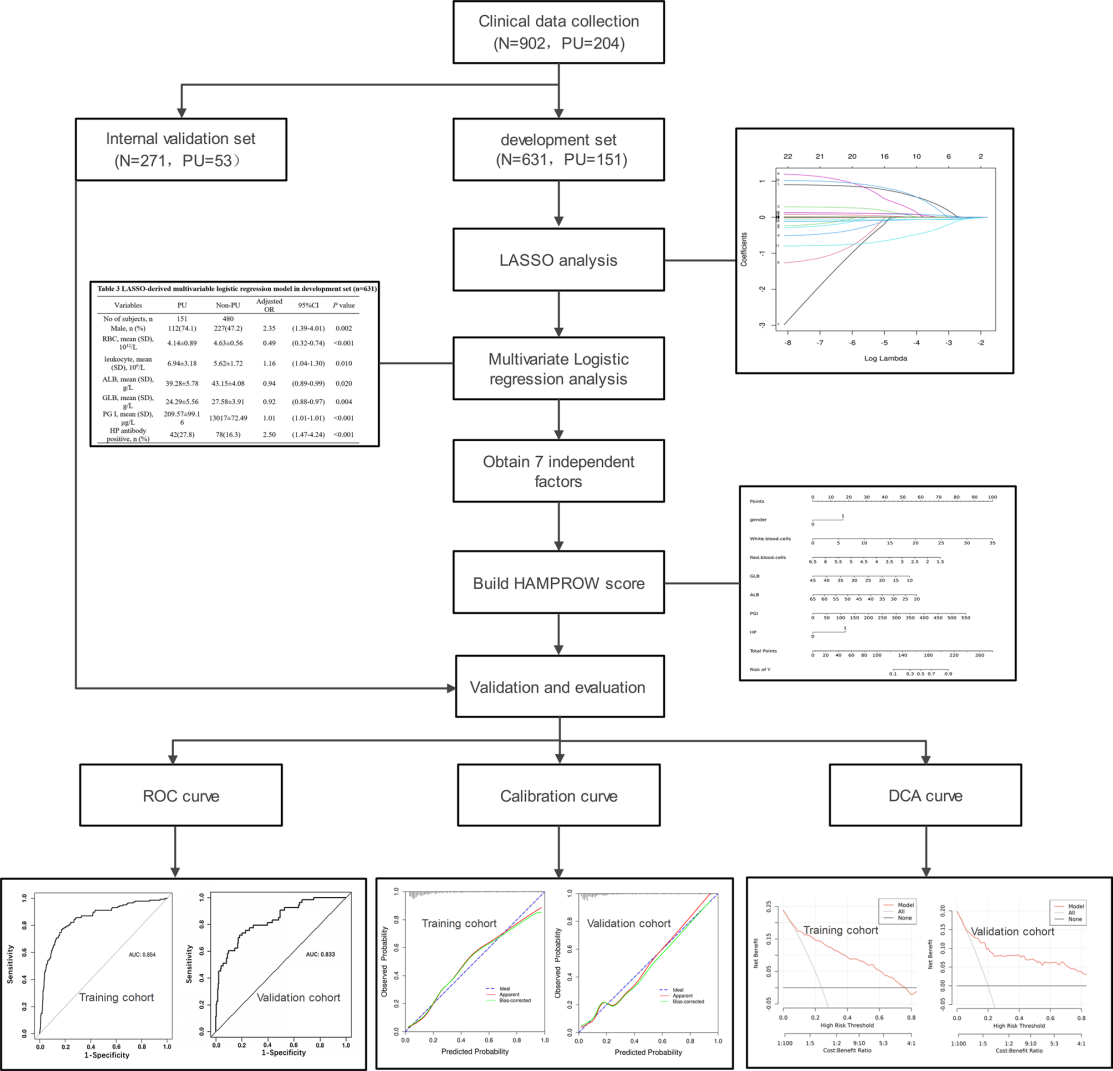
Flow chart of the study process. LASSO, the least absolute shrinkage and selection operator; ROC, receiver operating characteristic; DCA, decision curve analysis.

According to the formula presented by van Smeden et al.^15^ (the formula is available at https://mvansmeden.shinyapps.io/BeyondEPV/), we set the mean absolute prediction error (MAPE) to be no larger than 0.050, the anticipated occurrence rate of PU to be 0.2 and at most 8 candidate predictor parameters of the final model. Thus, at least 600 participants were included in the development cohort (Fig. 1). We set the sample ratio between the development and validation sets at 2:1. Therefore, a total of at least 900 participants were enrolled in this analysis.

### Model-building procedure and temporal validation

Among the enrolled patients, patients assessed between April 2019 and April 2021 were classified in the development cohort, and those assessed between May 2021 and May 2022 were classified in the validation cohort.

For the development cohort, least absolute shrinkage and selection operator (LASSO) regression was used to screen for risk factors, and factors with *P* < 0.05 were subsequently included in multivariate logistic regression analysis to determine the predictive factors of PU. Thus, a nomogram and receiver operating characteristic (ROC) curves were established based on the identified predictive factors. Discrimination and calibration of the established nomogram were determined by the area under the curve (AUC) and calibration curve, respectively. Decision curve analysis (DCA) was also conducted to assess the clinical practicability of the nomogram. Finally, temporal validation of the nomogram was performed in the validation set, and the area under the curve (AUC), sensitivity, specificity, positive predictive value (PPV) and negative predictive value (NPV) were calculated. Given that antiplatelet medication was always prescribed in the high-risk ASCVD population, the established nomogram was further validated in the population with high-risk ASCVD in the validation set.

### Statistical analysis

The quantitative variables in this study are presented as the means ± SDs or interquartile ranges, and the categorical variables are presented as proportions. Continuous variables were evaluated by t tests, categorical variables by Chi-square tests, and rank variables by independent U tests. All the statistical analyses were performed using SPSS version 26.0 (IBM, Armonk, New York) and R software, version 4.2.2, as well as MSTATA software (www.mstata.com).

## Results

### Participants

The flow chart is shown in Fig. 1. A total of 902 participants were included in this study, 12 of whom were excluded (2 due to acute upper gastrointestinal hemorrhage, 2 due to esophageal malignant tumors, and 8 due to inability to cooperate, resulting in incomplete imaging data). The age of the participants ranged from 18 to 87 years, with an average age of 53.8 ± 12.5 years. Among the participants, 468 (51.9%) individuals were male, 282 (31.3%) had hypertension, 56 (6.2%) had diabetes, 9 (1.0%) had a history of stroke, and 222 (24.6%) had high-risk ASCVD. According to the endoscopy results, there were 204 (22.6%) patients with peptic ulcers, including 89 (9.9%) with GU, 133 (14.7%) with DU, and 18 (2.0%) with both GU and DU. The baseline characteristics of all 902 participants are described in Table 1.

**Table 1:** Baseline characteristics of all subjects.

Variables	Entire cohort
No of subjects, n	902
Male, n (%)	468 (51.9)
Age, mean (SD), y	54.7 ± 14.8
Current smoking, n (%)	233 (25.8)
Hypertension, n (%)	282 (31.3)
Diabetes, n (%)	56 (6.2)
Stroke, n (%)	9 (1.0)
PU history, n (%)	23 (2.5)
High-risk ASCVD, n (%)	222 (24.6)
Antiplatelet use, n (%)	22 (2.4)
Anticoagulant use, n (%)	2 (0.2)
NSAIDs use, n (%)	15 (1.7)
HP antibody positive, n (%)	133 (19.6)
MLS, IQR	3 (1–4)
DU, n (%)	177 (14.7)
GU, n (%)	89 (9.9)
PU, n (%)	204 (22.6)

*SD* standard deviation, *NSAIDs* non-steroidal anti-inflammatory drugs, *HP* helicobacter pylori, *PU* peptic ulcer, *ASCVD* atherosclerotic cardiovascular disease, *MLS* modified Lanza score, *IQR* interquartile range, *DU* duodenal ulcer, *GU* gastric ulcer.

The development and validation sets were composed of 631 and 271 subjects, respectively, determined through time-based assignment. All the baseline characteristic data in the two sets are given in Table 2. A comparison of the baseline data showed that there was no significant difference between the development set and the validation set.

**Table 2:** Comparison of development and validation sets.

Variables	Development set	validation set	*P* value
No of subjects, n	631	271	
Male, n (%)	339 (53.7)	129 (47.6)	0.092
Age, mean (SD), y	53.8 ± 12.5	53.7 ± 12.3	0.875
Current smoking, n (%)	150 (23.8)	83 (27.9)	0.200
Hypertension, n (%)	197 (31.2)	85 (28.4)	0.966
Diabetes, n (%)	37 (5.8)	19 (7.0)	0.513
Stroke, n (%)	8 (1.3)	1 (0.4)	0.379
PU history, n (%)	15 (2.4)	8 (3.0)	0.616
Antiplatelet use, n (%)	15 (2.4)	7 (2.6)	0.854
Anticoagulant use, n (%)	2 (0.3)	0 (0)	1.000
NSAIDs use, n (%)	10 (1.6)	5 (1.8)	0.779
High-risk ASCVD, n (%)	158 (25.0)	64 (24.0)	0.670
WBC, mean (SD), 10^9^/L	5.93 ± 2.23	5.71 ± 2.07	0.160
RBC, mean (SD), 10^12^/L	4.51 ± 0.69	4.47 ± 0.66	0.409
Platelets, mean (SD), 10^9^/L	199.04 ± 61.39	196.41 ± 58.62	0.551
PT, mean (SD), s	11.57 ± 0.95	11.65 ± 0.91	0.220
APTT, mean (SD), s	31.99 ± 3.41	32.18 ± 3.80	0.472
INR, mean (SD),	1.05 ± 0.09	1.06 ± 0.08	0.129
ALT, mean (SD), U/L	23.93 ± 20.86	25.71 ± 31.26	0.395
GLB, mean (SD), g/L	26.79 ± 4.58	26.31 ± 4.18	0.145
ALB, mean (SD), g/L	42.22 ± 4.83	42.46 ± 4.85	0.489
Serum calcium, mean (SD), mmol/L	2.33 ± 2.90	2.21 ± 0.12	0.504
PG I, mean (SD), μg/L	149.17 ± 86.52	148.90 ± 83.54	0.966
PG II, mean (SD), μg/L	12.45 ± 11.53	12.02 ± 10.01	0.596
PG I/II, mean (SD)	15.14 ± 7.20	15.20 ± 7.58	0.898
G-17, mean (SD), pmol/L	10.27 ± 15.26	10.17 ± 14.88	0.900
PU, n (%)	151 (23.9)	53 (19.6)	0.150
HP antibody positive, n (%)	120 (19.0)	57 (21.0)	0.485

*PU* peptic ulcer, *OR* odds ratio, *CI* confidence interval, *SD* standard deviation, *NSAIDs* non-steroidal anti-inflammatory drugs, *RBC* red blood cell, *PT* prothrombin time, *APTT* active partial thromboplastin time, *INR* international standard ratio, *ALT* alanine aminotransferase, *GLB* globulin, *ALB* albumin, *PG I* pepsinogen I, *PG II* pepsinogen II, *G-17* gastrin-17, *HP* helicobacter pylori.

### Predictor selection and identification in the development set

Twenty-five characteristics measured at baseline (Table 1) were included in the LASSO regression analysis. After LASSO regression was performed (Supplementary Figure I), the following seven characteristics were found to be significant predictors of PU: male sex, white blood cell (WBC) count, red blood cell (RBC) count, globulin (GLB), albumin (ALB), PGI and HP antibody positivity. As shown in Supplementary Figure II, the ROC analysis of the abovementioned variables yielded AUC values greater than 0.5 for male sex (AUC = 0.631), WBC (AUC = 0.654), RBC (AUC = 0.667), GLB (AUC = 0.694), ALB (AUC = 0.703), PGI (AUC = 0.759), and HP antibody positivity (AUC = 0.550). Further multivariate logistic regression analysis revealed that male sex, antiplatelet agent use, WBC count, RBC count, GLB, PGI and HP antibody positivity were independently associated with the presence of PUs (all *P* < 0.05; see Supplementary Table I).

### Development of the nomogram for PU prediction

The risk of PUs was calculated using a multivariate logistic regression equation: ln(P/1−P) = 3.92103 + 0.85315*(male) + 0.14551*(WBC) −0.72291*(RBC) −0.07839*(GLB) −0.06550*(ALB) + 0.00788*(PGI) + 0.91716*(HP antibody-positive). In this equation, P represents the probability of a PU.

**Figure 2:**
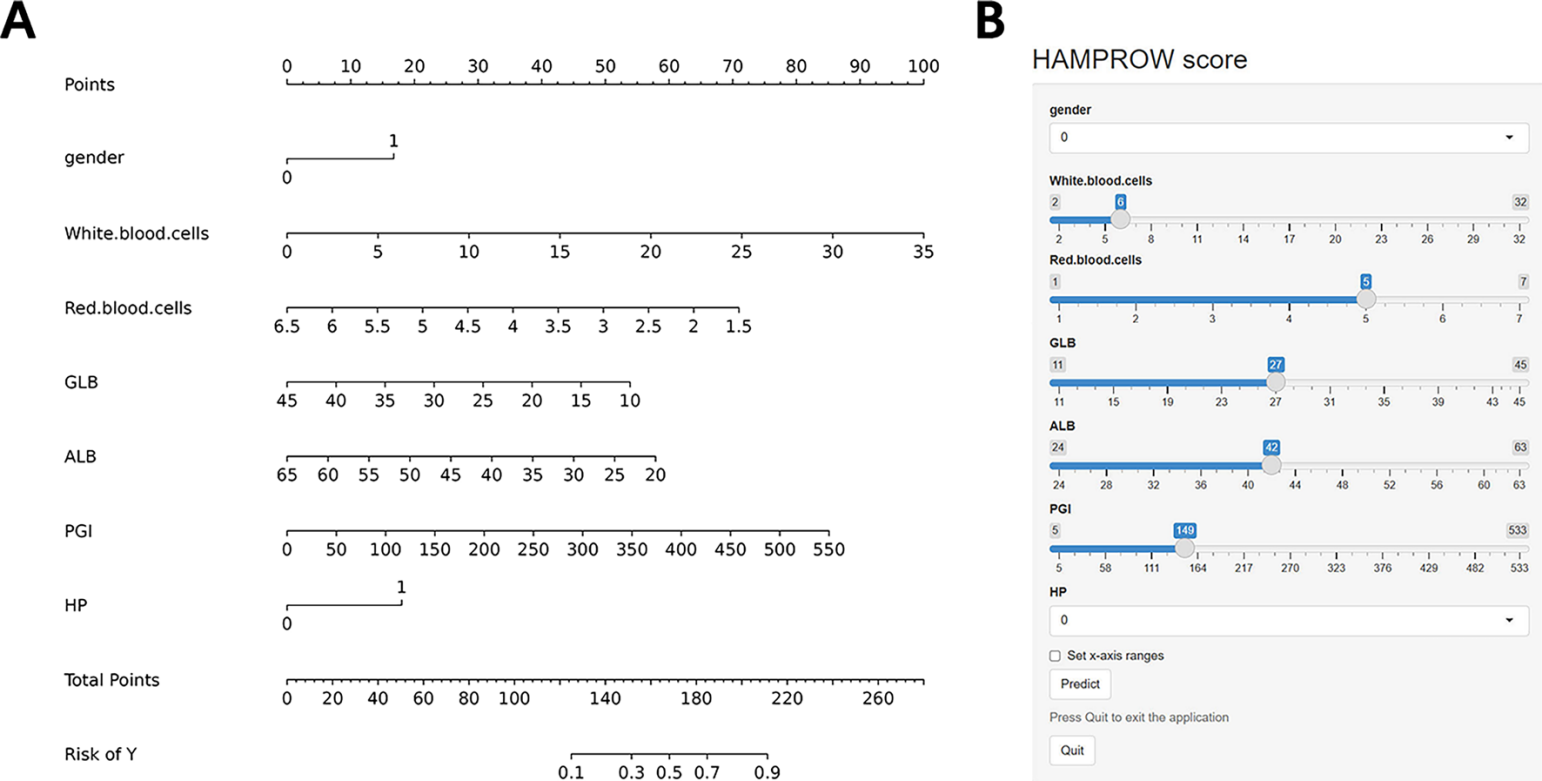
The nomogram HAMPROW score. (**A**) To use the nomogram, draw an upward vertical line from each covariate to the points bar to calculate the number of points. Based on the sum of the covariate points, draw a downward vertical line from the total points line to calculate the probability of developing PU. (**B**) Online dynamic nomogram accessible athttps://worldn.shinyapps.io/dynnomapp/. GLB, globulin; ALB, albumin; PG I, pepsinogen I; HP, helicobacter pylori.

A predictive nomogram for PUs [hereafter referred to as the HAMPROW (HP antibody, Albumin, Male, PGI, RBC, globulin, WBC)] (Fig. 2) was developed based on the results of multivariate logistic regression, which is illustrated in Fig. 2A; the nomogram is available online (https://worldn.shinyapps.io/dynnomapp/) and presented in Fig. 2B. Factors were assigned points according to the absolute maximum beta value from the logistic regression model. Due to the differences in units, points were assigned differently for continuous predictors (RBC, WBC, ALB, GLB, and PGI) and categorical predictors (male, HP antibody-positive). The length of the line on the nomogram reflects the impact of these factors on PU, with a longer line indicating a greater impact. By summing all the points, a total score can be obtained, which helps determine the risk of PU by locating it on the HAMPROW score during subject presentation.

**Figure 3:**
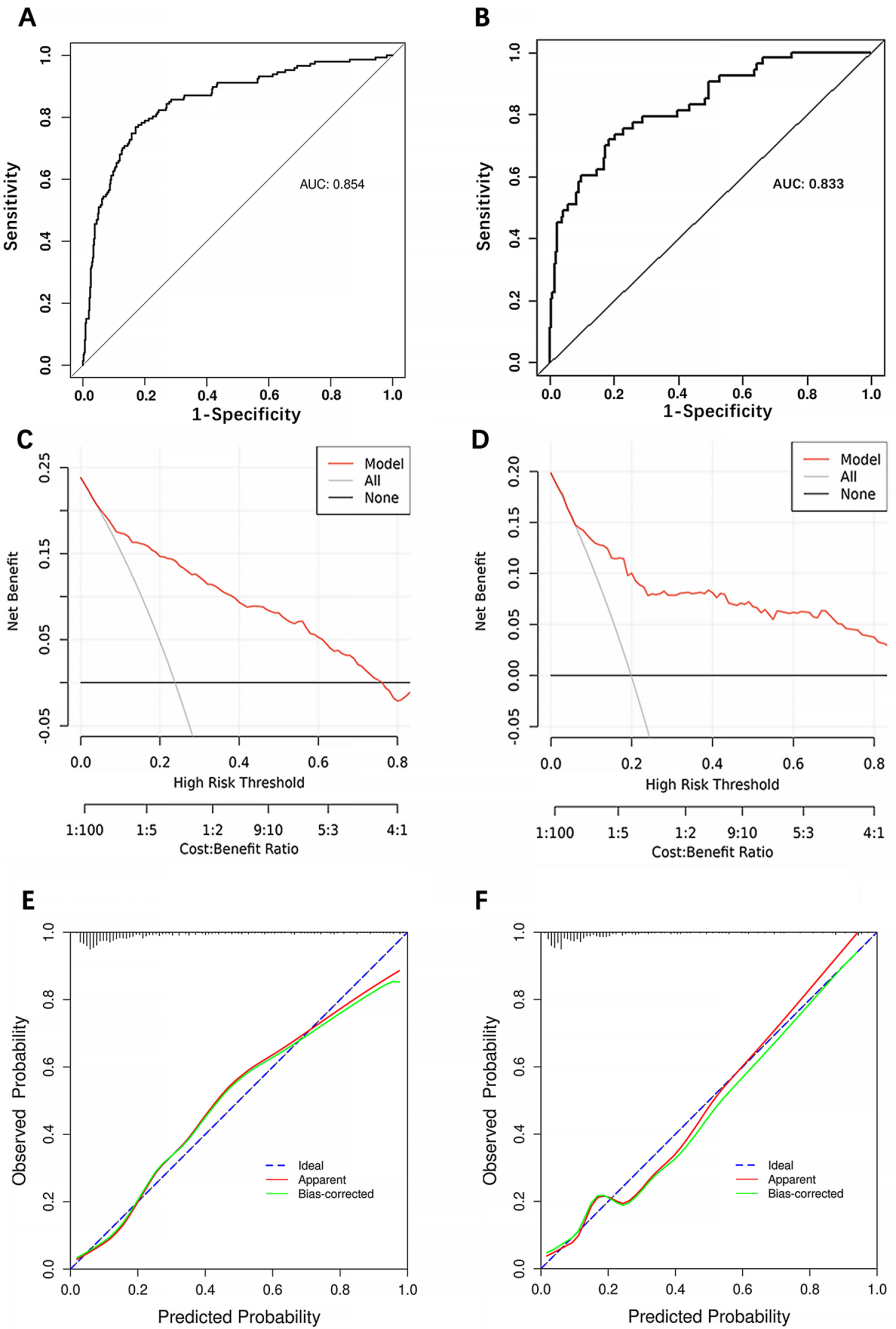
Evaluation of validity, reliability, and decision curve of HAMPROW score. (**A**) ROC curve for the HAMPROW score based on the development set. The bias-corrected AUC is 0.854. (**B**) ROC curve from the validation set and the AUC is 0.833. (**C**) Calibration curves of the HAMPROW score for the development set. (**D**) Calibration curves of the HAMPROW score for the validation set (**E**) Decision curve analysis of the HAMPROW score of the development set. (**F**) Decision curve analysis of the HAMPROW score of the validation set. AUC, area under the curve; ROC, receiver operating characteristic.

ROC curve analysis revealed that the predictive value of the HAMPROW score for PU in the development cohort was good, with an AUC of 0.854 (95% CI = 0.816–0.891) (Fig. 3A). The cutoff score of the HAMPROW was 146, with a sensitivity of 76.9% and specificity of 83% (Youden index = 0.599).

### Validation of the HAMPROW score

We further tested the predictive value of the HAMPROW score in the validation cohort (n = 271). According to the ROC curve analysis, the area under the curve (AUC) was 0.833 (95% CI = 0.771–0.895) (Fig. 3B), which was significantly greater than that of any factor in the HAMPROW (all *P* < 0.05) (Supplementary Figure III). Moreover, the area under the curve (AUC) of the HAMPROW score was not significantly different between the development and validation sets (*P* > 0.05). Additionally, calibration curves and decision curve analysis (DCA) were also plotted to show the clinical utility of the HAMPROW score (Fig. 3C–F). The nomogram exhibited a net benefit across a wide range of threshold probabilities for predicting PUs, regardless of the development set or the validation set. A cutoff value of 146 for the HAMPROW score had a sensitivity of 74.5% and specificity of 79.3%. The PPV was 46.9%, and the NPV was 92.7%.

In the population with high-risk ASCVD in the validation set (n = 63), the AUC of the HAMPROW score was 0.840 (95% CI = 0.723–0.957). A cutoff value of 146 for the HAMPROW score had a sensitivity of 82.4% and specificity of 71.1%. The PPV was 51.9%, and the NPV was 88.9% in this subgroup.

## Discussion

In this study, we constructed a HAMPROW score for predicting PUs using seven indicators: HP antibody concentration, albumin concentration, male sex, PGI, RBC, globulin concentration, and WBC count. Our novel nomogram (HAMPROW) demonstrated excellent predictive performance in both the development and validation sets, providing a convenient and reliable method for early screening of the PU risk population.

Of the seven variables associated with the HAMPROW, consistent with previous findings, we proved that male sex^16^, a history of HP infection^17^, and RBC^18^ were key factors associated with the presence of PUs. In addition, this study further identified four other factors from blood tests that are involved in the occurrence of PU: excessive secretion of gastric acid, decreased repair ability of the gastrointestinal mucosa (i.e., PGI) and impaired gastrointestinal mucosal barrier function (i.e., WBC, ALB and GLB).

The serum PGI, initially discovered by Samloff IM^19^, has been found to accurately indicate gastric acid levels and serve as a reliable biomarker for the noninvasive assessment of gastric mucosal secretion^7,20,21^. Patients with PU exhibit elevated PGI levels^22,23^, while those with gastric atrophy exhibit significantly reduced PGI levels^24,25^, indicating its potential as a biomarker for both secretory function and the state of the gastric mucosa. Gastric acid can inflict direct damage on the upper gastrointestinal mucosa through chemical stimulation and induce secondary harm by disrupting mucosal defense and repair mechanisms^26,27^. Increased gastric acid secretion plays an important role in the development of upper gastrointestinal mucosal damage. Notably, antiplatelet medications can also increase gastric acid secretion, resulting in exacerbation of gastrointestinal mucosal damage. Studies have confirmed that increased gastric acid secretion following antiplatelet therapy is a crucial risk factor for upper gastrointestinal bleeding ^28,29^.

Our study also highlights the important screening potential of WBC, serum ALB, and GLB levels for detecting the presence of PUs. Although all three factors reflect compromised gastrointestinal mucosal barrier function, they were not mutually adjusted for in the multivariate analysis, suggesting that they play independent roles in promoting PU development. WBCs commonly serve as markers for assessing inflammatory status or blood concentration. Elevated WBC counts may indicate inflammation or immune response activation, potentially leading to mucosal barrier damage and facilitating PU development. GLB actively contributes to immune function and antibody production and is vital for maintaining a healthy immune system. Changes in GLB levels during PU development signify shifts in immune function, mirroring mucosal barrier changes. Conversely, the serum ALB concentration reflects patient nutritional status. Previous research has indicated that prolonged mucosal injury in the upper gastrointestinal tract leads to impaired nutrient absorption and decreased albumin levels^30^. Furthermore, low serum ALB concentrations have been identified as a predictor of unfavorable treatment outcomes in patients with PU, indicating heightened severity of gastric mucosal barrier damage among these individuals^31^.

Notably, NSAIDs are not represented in the HAMPROW score. Although previous studies have identified NSAIDs as potential risk factors for PU, our study, through both univariate and multivariate analyses, revealed no associations between PU and either antiplatelet drug use or non-aspirin NSAIDs. The primary contributing factor to this disparity is the relatively low prevalence of non-aspirin NSAID use among healthy individuals, with only 2.4% utilizing antiplatelet drugs (development set: 2.4%, validation set: 2.6%) and non-aspirin NSAID usage accounting for 1.7% (development set: 1.6%, validation set: 1.8%) of the overall population. Prior studies that emphasized NSAIDs as a risk factor focused on populations with existing PUs, potentially resulting in differences in inclusion criteria.

Although each variable can be used to predict PU, the HAMPROW score generated from these seven variables demonstrated superior effectiveness in predicting the occurrence of PU compared to that of individual variables alone. In our current study, the PPV of the HAMPROW was low (46.9%), implying that more than half of the individuals with a HAMPROW score higher than 146 are likely to have false-positives. This highlights the necessity for further screening to confirm the presence of PUs. The diminished PPV may be attributed to the relatively low incidence of PUs in the physical population (approximately 22.6%). However, the NPV of the score was high (92.7%). A HAMPROW score less than 146 signifies an extremely low risk of PU occurrence, indicating that antiplatelet therapy is safe for such patients. Additionally, the HAMPROW score showed a similar predictive ability in the ASCVD subgroup population as in the overall validation cohort (AUC _validation set_ *vs.* AUC _high-risk ASCVD population_: 0.833 *vs*. 0.840). This result suggests that the HAMPROW score can be applied not only to the general population undergoing physical examination but also to those requiring antiplatelet therapy due to the high risk of ASCVD.

The strengths of our model, which support the validity of our findings, include the simplicity of a statistical model comprising a limited number of clinically relevant predictors and the consistency of the results with those of all considered studies. However, there are still some limitations in our study. First, the data used in this study were obtained from a single center in China, possibly limiting the generalizability of the nomogram to patients worldwide. Further studies using data from multiple centers are needed to validate our model externally. Second, additional research is needed to explore the clinical application of the identified biomarkers. It is important to investigate whether applying our predictive nomogram for risk stratification in patients receiving antiplatelet therapy with gastric mucosal protection can effectively reduce the incidence of gastrointestinal bleeding and adverse reactions. Third, some items related to the occurrence of PUs, such as alcohol consumption history, *Helicobacter pylori* eradication history, and gastrointestinal symptoms, were not recorded. This may have resulted in the omission of some PU-related risk factors. Future studies should address these issues to refine the selection of variables for collection.

In conclusion, we developed a quantifiable, user-friendly, and reliable predictive nomogram, the HAMPROW score, for PU screening in a population subjected to physical examination. Future research will focus on confirming the value of the HAMPROW score in guiding antiplatelet therapy through prospective validation methods.

### Supplementary Information

Supplementary Information.

## Data Availability

All datasets generated and/or analyzed during the current study available from the corresponding author on reasonable request.
