# Supplementary material for: Assessing peptic ulcer risk with the HAMPROW score in the general Chinese population
Source: Sci Rep. 2024 Feb 23;14:4442. doi: 10.1038/s41598-024-55224-0 (PMC10891164; doi:10.1038/s41598-024-55224-0)
Supplement: Supplementary file 1 — Supplementary Information. [file 41598_2024_55224_MOESM1_ESM.pdf]

Supplementary Files

Supplementary Table I. LASSO-derived multivariable logistic regression model for predicting PU in development set (n=631).

Variables	PU	Non-PU	Adjusted OR	95%CI	*P* value
No of subjects, n	151	480			
Male, n (%)	112(74.1)	227(47.2)	2.35	(1.39-4.01)	0.002
RBC, mean (SD), 10^{12}/L	4.14±0.89	4.63±0.56	0.49	(0.32-0.74)	<0.001
WBC, mean (SD), 10^{9}/L	6.94±3.18	5.62±1.72	1.16	(1.04-1.30)	0.010
ALB, mean (SD), g/L	39.28±5.78	43.15±4.08	0.94	(0.89-0.99)	0.020
GLB, mean (SD), g/L	24.29±5.56	27.58±3.91	0.92	(0.88-0.97)	0.004
PG I, mean (SD), μg/L	209.57±99.16	13017±72.49	1.01	(1.01-1.01)	<0.001
HP antibody positive, n (%)	42(27.8)	78(16.3)	2.50	(1.47-4.24)	<0.001

PU, peptic ulcer; OR, odds ratio; CI, confidence interval; SD, standard deviation; RBC, red blood cell; GLB, globulin; PG I, pepsinogen I; HP, helicobacter pylori.

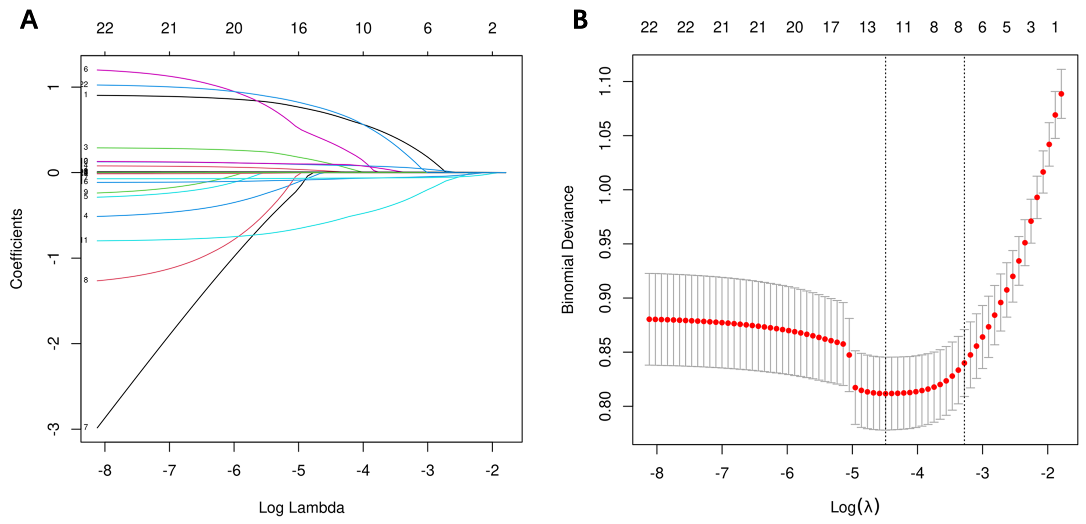

Supplementary Figure I. Feature selection using the least absolute shrinkage and selection operator (LASSO) binary logistic regression model. (A) LASSO coefficient profiles of the 22 baseline characteristics. (B) Tuning parameter (λ) selection in the LASSO model used 10-fold cross-testing via minimum criteria.

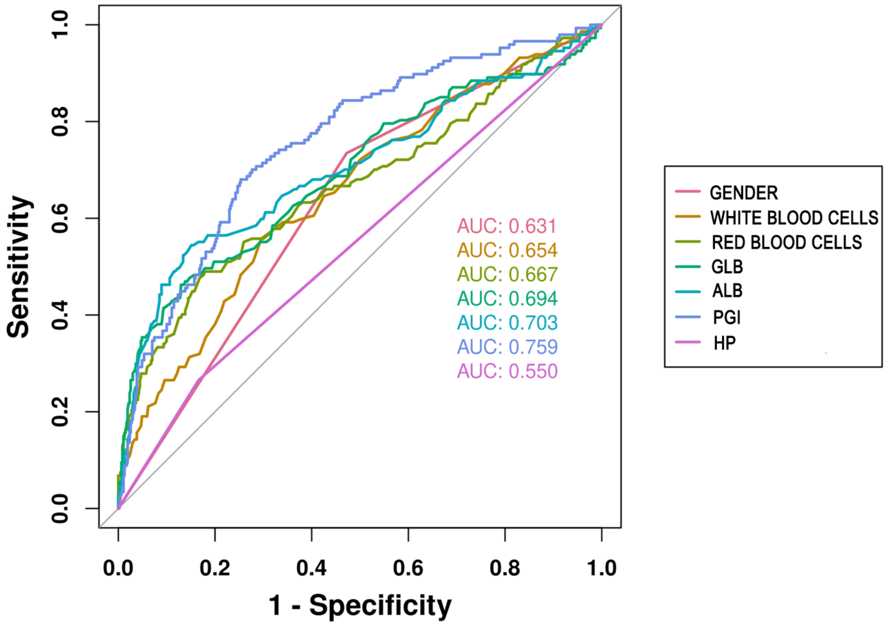

Supplementary Figure II. ROC curve analysis of seven predictors in development sets. ROC, receiver operating characteristic; AUC, area under the curve; GLB, globulin; ALB, Albumin; PGI, pepsinogen I; HP, helicobacter pylori.

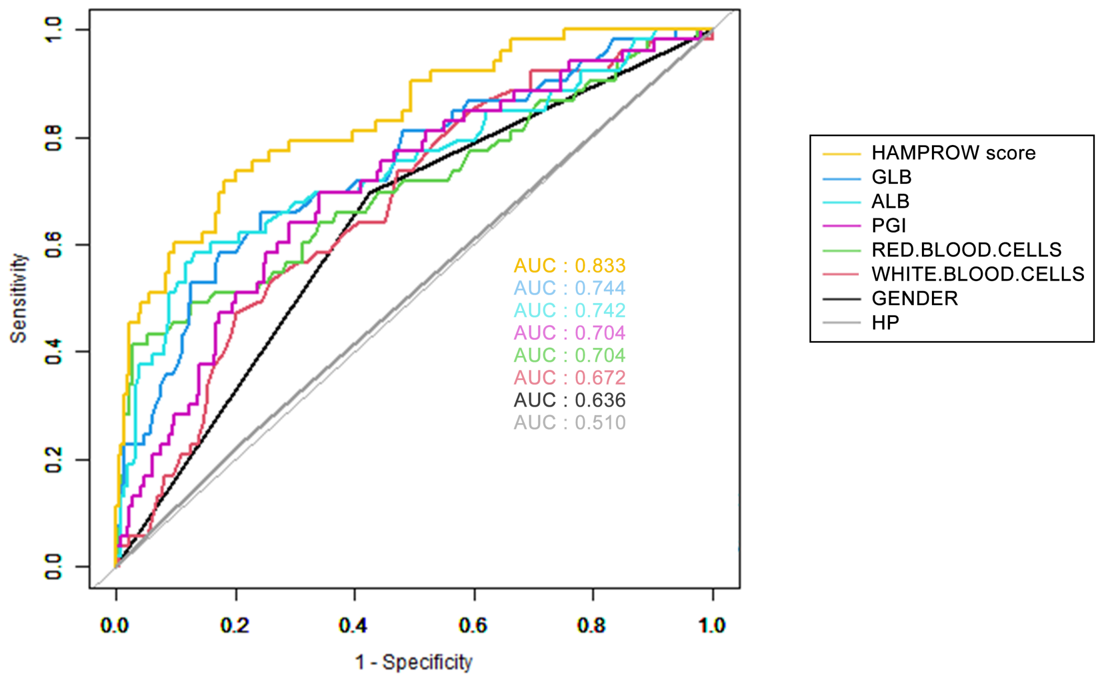

Supplementary Figure III. Comparison of HAMPROW Score and Seven Factors using ROC Analysis in Validation Sets. ROC, receiver operating characteristic; AUC, area under the curve; GLB, globulin; ALB, albumin; PGI, pepsinogen I; HP, helicobacter pylori.
